# A Comparative Survey of the Frequency and Distribution of Polymorphism in the Genome of *Xenopus tropicalis*

**DOI:** 10.1371/journal.pone.0022392

**Published:** 2011-08-04

**Authors:** Chris Showell, Samantha Carruthers, Amanda Hall, Fernando Pardo-Manuel de Villena, Derek Stemple, Frank L. Conlon

**Affiliations:** 1 UNC McAllister Heart Institute, University of North Carolina at Chapel Hill, Chapel Hill, North Carolina, United States of America; 2 Department of Genetics, University of North Carolina at Chapel Hill, Chapel Hill, North Carolina, United States of America; 3 Carolina Center for Genome Sciences, University of North Carolina at Chapel Hill, Chapel Hill, North Carolina, United States of America; 4 Department of Biology, University of North Carolina at Chapel Hill, Chapel Hill, North Carolina, United States of America; 5 Wellcome Trust Sanger Institute, Wellcome Trust Genome Campus, Cambridge, United Kingdom; Duke University, United States of America

## Abstract

Naturally occurring DNA sequence variation within a species underlies evolutionary adaptation and can give rise to phenotypic changes that provide novel insight into biological questions. This variation exists in laboratory populations just as in wild populations and, in addition to being a source of useful alleles for genetic studies, can impact efforts to identify induced mutations in sequence-based genetic screens. The Western clawed frog *Xenopus tropicalis* (*X. tropicalis*) has been adopted as a model system for studying the genetic control of embryonic development and a variety of other areas of research. Its diploid genome has been extensively sequenced and efforts are underway to isolate mutants by phenotype- and genotype-based approaches. Here, we describe a study of genetic polymorphism in laboratory strains of *X. tropicalis*. Polymorphism was detected in the coding and non-coding regions of developmental genes distributed widely across the genome. Laboratory strains exhibit unexpectedly high frequencies of genetic polymorphism, with alleles carrying a variety of synonymous and non-synonymous codon substitutions and nucleotide insertions/deletions. Inter-strain comparisons of polymorphism uncover a high proportion of shared alleles between Nigerian and Ivory Coast strains, in spite of their distinct geographical origins. These observations will likely influence the design of future sequence-based mutation screens, particularly those using DNA mismatch-based detection methods which can be disrupted by the presence of naturally occurring sequence variants. The existence of a significant reservoir of alleles also suggests that existing laboratory stocks may be a useful source of novel alleles for mapping and functional studies.

## Introduction

The Western clawed frog *Xenopus tropicalis* (*X. tropicalis*) has enormous potential to enhance our understanding of the molecular control of embryonic development and the evolution of biological pathways [1]. It is closely related to the South African clawed frog *Xenopus laevis* (*X. laevis*), and shares its many advantages as a model for developmental biology. The recent publication of the genome sequence of *X. tropicalis* has highlighted the similarities between its genes and those of humans, including extensive conservation of the synteny relationships between genes, in spite of the large evolutionary distance between the species [2]. The *X. tropicalis* genome contains orthologs of at least 1,700 human genes known to be involved in disease and therefore the frog will be a valuable biomedical model in the future, particularly for studies of congenital diseases. In embryos of both *X. laevis* and *X. tropicalis*, gene function can be inhibited by microinjection of morpholino oligonucleotides that block translation or splicing of specific messenger RNAs but, being a diploid species with a shorter generation time, *X. tropicalis* presents the opportunity to combine what we know of its embryonic development with genetic analysis. The isolation of alleles that harbor functionally significant sequence variation is an essential step in this approach and this can be achieved by screening either for sequence variation (or polymorphism) that exists naturally within populations or for novel mutations, the frequency of which can be dramatically increased by chemical or radiological mutagenesis [3].

Genetic polymorphism within populations underlies the phenotypic variation that allows for evolutionary adaptation of species. Naturally occurring alleles segregating within a population can have beneficial or detrimental effects on gene function and organism fitness. For example, a number of polymorphisms found in stocks of the most commonly used laboratory strain of *X. tropicalis* have been shown to result in developmental abnormalities when homozygous. The genes affected by the natural mutations *bubblehead*, *curly* and *grinch* are yet to be determined but the dysfunctional alleles disrupt a variety of developmental processes including the development of craniofacial structures, the gut, axial structures, and the ear [4]. While these polymorphisms were identified on the basis of their resultant mutant phenotypes, it is also possible to identify novel naturally occurring polymorphisms using tilling strategies – a process known as ‘ecotilling’. In other organisms, this approach has been used successfully to screen for variation in out-bred populations [5], [6], [7], [8], [9]. Studies of the naturally occurring mutants *bubblehead*, *curly* and *grinch* demonstrate that naturally occurring sequence variation may represent a valuable reservoir of alleles that can be used for functional studies of genes. What other mutants might be harbored by the existing laboratory strains? Assaying the frequency and distribution of polymorphism across the *X. tropicalis* genome is one way to determine whether the natural mutants discovered so far are likely to be rare anomalies or just the first examples of more widespread genetic and phenotypic variation that could be harnessed to provide useful alleles for study.

While the *X. tropicalis* genome has been extensively sequenced, the data generated represents the genome of just a single seventh-generation inbred Nigerian frog [2]. Up to now, the only systematic survey of polymorphism in *X. tropicalis* has been an effort to identify SSLPs between Nigerian frogs and one of two strains originating from Ivory Coast, for use in gene mapping [10], [11]. Consequently, the extent of genetic variation within strains has not been examined. In the course of studies testing mutagenesis techniques and mismatch-based mutation detection in *X. tropicalis*, we encountered an unexpectedly high frequency of polymorphism in our laboratory-bred frogs. Measurement of the frequency and distribution of this variation is important because the existing outbred strains are the basis of current studies and of future genetic resources, including inbred lines. Also, polymorphism in regions of genes targeted in tilling screens can significantly interfere with the detection of induced mutations by mismatch-based methods such as CelI endonuclease digestion. These methods remain important for genotype-based mutation screening, even in the age of next-generation sequencing, because they are suited to screening large numbers of individuals for mutations in specific target genes. So, knowledge of the frequency of polymorphism is important for the design of this type of genetic screen.

The frequency of polymorphism in laboratory strains is determined by two factors – the original frequency of polymorphism in the wild-caught founders of the strain, and the number of generations of inbreeding that produced the current stocks. While we know the second factor, the first is unknown. Therefore, to know the polymorphism frequency within laboratory strains, it must be measured directly. Here, we describe novel sequence variants identified in sequencing-based screens of a panel of developmental genes. We assess both the frequency and type of natural polymorphism in the most widely used laboratory strain of *X. tropicalis*, originating from Nigeria, and in a strain originating from Ivory Coast. The utility of existing strains for identifying novel mutants and genetic markers is discussed, together with the implications of extensive sequence polymorphism for mismatch-based mutation screens. In addition, we quantify the rate of genotyping errors due to allelic dropout (a failure to amplify one allele from a heterozygous individual) in sequence-based genotyping, a factor that can affect the results of PCR-based efforts to map mutations using microsatellites or other polymorphic markers.

## Results

### DNA sequencing identifies frequent polymorphism in Nigerian strain *X.tropicalis*

Conventional Sanger dideoxy terminator sequencing was used to enable the sequencing of a panel of developmental gene amplicons from a large number of individuals derived from crosses or from laboratory populations. This allows the detection of variation in expected Mendelian ratios where appropriate, gives an indication as to whether alleles are common within a population or breeding stock, and is valuable in determining whether sequence variants correspond to distinct alleles or are derived from errors in PCR amplification or base-calling. To identify and characterize natural sequence variants, we screened two groups of F1 tadpoles (‘Group 1’ and ‘Group 2’, offspring from two independent crosses of Nigerian strain F5 frogs) by direct sequencing of a panel of 23 amplicons corresponding to regions from 17 genes (see Supplementary Information S1). The genes sequenced are involved in a variety of developmental processes including neurogenesis, cardiogenesis, mesoderm specification and embryonic patterning. They encode factors involved in transcriptional regulation and intercellular signaling, and are widely distributed in the *X.tropicalis* genome, with each gene found on a unique genomic scaffold in genome assembly v4.1. PCR amplicons were chosen based on their polyG∶C base pair content, with preference for stretches of three G∶C base pairs and longer, from a larger panel of amplicons developed for sequence-based mutation screening [3]. Collectively, the amplicons correspond to 4,907 bp of coding sequence and 4,472 bp of non-coding sequence. We generated PCR products from a total of 384 individuals, sequenced them directly and screened the resulting traces for heterozygous base positions and insertions/deletions (indels). We also looked for sequence variation between the two independent F1 sibling groups, as these may carry distinct alleles. For the purposes of this study we defined an individual polymorphism as being a variation arising from a discrete mutation event, i.e. either a single variant nucleotide or a single contiguous stretch of inserted or deleted nucleotides. Contiguous stretches of several variant nucleotides constitute multiple polymorphisms derived from independent mutation events. We identified 16 polymorphisms in ten amplicons from ten genes (Tables 1 and 2). These were identified within the sibling groups (either Group 1 or Group 2), with no further polymorphisms found between groups. The alleles and genotype frequencies in the datasets were determined. To independently verify the polymorphisms, the amplicon set was amplified from parental DNA and sequenced. The results of parental genotyping confirmed all of the polymorphisms detected and agreed with the predicted parental genotypes. Aside from these polymorphisms, we found no instances of individual F1 tadpoles carrying unique variants that might have arisen from mutations in the parental germlines.

**Table 1:** Polymorphisms identified in UNC Nigerian F5 Group 1.

Gene	#bp screened	Sequence variants	Class	Genotype Ratio	Expected Genotype Ratio
cdx4	389 bp	a)CTATCaAACAT b)CTATCgAACAT	Silent (non-coding)	10∶60∶11 (g/g ∶ a/g ∶ a/a)	0∶1∶0
chrd	326 bp	a)GTAACatAGTTT…CAGGGtGTACA b)GTAAC–AGTTT…CAGGGaGTACA	Silent (non-coding)	50∶30∶2 (at/at t/t ∶ –/at a/t ∶ –/– a/a)	1∶1∶0
hhex	431 bp	a)ATTACtTAT(tAAACA…AACaATT) c)ATTACaTAT(tAAACA…AACaATT)	Silent (non-coding)	61∶16∶6b (t/t ∶ a/t ∶ a/a)	1∶1∶0
noggin1	480 bp	a)(TGAAcCCCCCCAAT)…CTCCcCTGA b)(TGAAcCCCCCCAAT)…CTCCtCTGA	Silent (non-coding)	6∶61∶7 (t/t ∶ c/t ∶ c/c)	1∶1∶0
oct1	423 bp	a)CTTTGtttgTATGAT b)CTTTG----TATGAT	Silent (non-coding)	4∶33∶39 (----/---- ∶ ----/tttg ∶ tttg/tttg)	0∶1∶1
pax2 (amplicon 2)	484 bp	a)(TTTaGTC…)TTA-TTT…GACcCCA c)(TTTaGTC…)TTAtTTT…GACtCCA	Silent (non-coding)	39∶33∶4 (t/t t/t ∶ t/- t/c ∶-/- c/c)	1∶1∶0
pax8	392 bp	a)TGGAAaGAACA b)TGGAAgGAACA	Silent (non-coding)	45∶39∶1 (g/g ∶ g/a ∶ a/a)	1∶1∶0

Data from direct sequencing of Group 1 individuals are summarized. Polymorphic nucleotides are indicated in lower case, with flanking and intervening nucleotides in upper case. Nucleotides absent from an allele (i.e. indel polymorphism) are represented by an equivalent number of dashes. Where a single SNP, multi-nucleotide polymorphism or indel polymorphism was found, the variants are shown within the same sequence expression. For amplicons with more than one polymorphic region, the genotype of each allele is listed individually. Where additional polymorphic regions were found in other datasets but were not polymorphic in the dataset in question, the genotypes at the additional variant positions are shown in parentheses. Primer regions were excluded when determining the number of base pairs screened for each amplicon. The positions of the polymorphic nucleotides are given as positions within the full amplicon sequence, including primer regions. Full genotype ratios recovered in the dataset are given for all genes except hhex, where two genotypes arising from allelic dropout are omitted for brevity. Abbreviations: bp, base pairs; cds, coding sequence; nt, nucleotide; UTR, untranslated region.

**Table 2:** Polymorphisms identified in UNC Nigerian F5 Group 2.

Gene	#bp screened	Sequence variants	Class	Genotype Ratio	Expected Genotype Ratio
cdx4	389 bp	a)CTATCaAACAT b)CTATCgAACAT	Silent (non-coding)	5∶56∶76 (g/g ∶ g/a ∶ a/a)	0∶1∶1
chrd	326 bp	a)GTAACatAGTTT…CAGGGtGTACA b)GTAAC–AGTTT…CAGGGaGTACA	Silent (non-coding)	126∶153∶5 (at/at t/t ∶ –/at a/t ∶ –/– a/a)	1∶1∶0
frzd7	439 bp	a)CCTCTaTGcACGGA b)CCTCTgTGtACGGA c)CCTCTgTGcACGGA	Silent (Leu/Leu43 and Cys/Cys44)	2∶130∶121∶7∶4 (g/g t/t ∶ a/g c/t ∶ a/g c/c ∶ a/a c/c ∶ g/g c/c)	0∶1∶1∶0∶0
gata6 (amplicon 2)	456 bp	a)TTGGCCaGCTGG b)TTGGCCtGCTGG	Silent (Pro/Pro278)	137∶135∶4 (t/t ∶ a/t ∶ a/a)	1∶1∶0
hhex	431 bp	a)ATTACtTATtAAACA…AACaATT b)ATTACaTATcAAACA…AACtATT c)ATTACaTATtAAACA…AACaATT	Silent (non-coding)	66∶64∶60∶70 (t/t t/t a/a ∶ a/t c/t a/t ∶ a/t t/t a/a ∶ a/a c/t a/t)	1∶1∶1∶1
noggin1	480 bp	a)CCTCCcCTGAT b)CCTCCtCTGAT	Silent (non-coding)	9∶199∶12 (t/t ∶ c/t ∶ c/c)	0∶1∶0
pax2 (amplicon 2)	484 bp	b)TTTcGTC…(TTAtTTT)…(GACtCCA) c)TTTaGTC…(TTAtTTT)…(GACtCCA)	Silent (non-coding)	2∶137∶140 (c/c ∶ c/a ∶ a/a)	0∶1∶1
pax6 (amplicon 3)	433 bp	a)(TGActaagtcattgaGGA)…GAG—AAA b)(TGActaagtcattgaGGA)…GAGgagAAA	Deletion (Gly186)	5∶127∶143 (—/— ∶ —/gag ∶ gag/gag)	0∶1∶1
pax8	392 bp	a)TGGAAaGAACA b)TGGAAgGAACA	Silent (non-coding)	132∶136∶5 (g/g ∶ g/a ∶ a/a)	1∶1∶0

Data from direct sequencing of Group 2 individuals are summarized. See the legend accompanying Table 1 for a detailed description of the allele notation system used.

The majority of the polymorphisms in Groups 1 and 2 are silent, because they either encode synonymous codons or are located in non-coding regions. An exception is the three nucleotide indel found within the coding sequence of *pax6*. These nucleotides do not represent a discrete codon, but the alleles encode protein variants that differ by the presence or absence of a glycine residue at position 186. Only heterozygotes and individuals homozygous for the allele containing these three nucleotides (the ‘insertion’ allele) were detected reliably amongst the Group 2 siblings, with the same genotypes detected in their parents. Similarly, Group 1 and parental samples were homozygous for the insertion allele. Therefore, whether the deletion allele would be deleterious to pax6 function when homozygous was not determined. The affected amino acid is located between the paired box and homeodomain DNA-binding domains, within a short glycine homopolymer stretch that is not highly conserved amongst orthologs in other vertebrates.

Two polymorphisms detected in Group 1 had unexpected genotype distributions. With respect to the polymorphism in *hhex*, the parents of this group were found to be a heterozygote (carrying both the ‘a’ and ‘t’ alleles detailed in Table 1) and a homozygote carrying only the ‘t’ allele. In this case, the progeny were predicted to be of the same genotypes as their parents, with heterozygotes and homozygotes appearing in approximately equal numbers. However, even assuming that the homozygous ‘a’ individuals were mis-genotyped heterozygotes (see below), the homozygotes greatly outnumbered the heterozygotes with a ratio of 61∶22. A similarly skewed distribution was observed when Group 1 individuals were genotyped for the polymorphism detected in *noggin1*, where the parents were again found to be a heterozygote and a homozygous ‘t’ individual. Here, when mis-genotyped samples were taken into account, heterozygotes outnumbered homozygotes with a ratio of 68∶6. We applied the Chi-square test to the *hhex* and *noggin1* genotype datasets and found statistically significant p-values of <0.0003 and <0.0001 respectively (*hhex* Chi-square values = 16.496 and 18.328; *noggin1* Chi-square value = 50.284). These polymorphisms were found to be in non-coding regions of the genes. Interestingly, Group 2 also carried the *hhex* polymorphism at nt73, but there appeared to be no lethality associated with the ‘a’ allele in this group (see Table 2). These data suggest that the skewed genotype distributions may result from deleterious alleles at loci linked to *hhex* and *noggin1*.

In total, we screened 3,496,104 base pairs for polymorphisms and mutations across the 23 selected amplicons. When the polymorphisms detected in each group were collated, the frequencies of polymorphism within the amplicon panel were 0.00096 and 0.0014 for Groups 1 and 2 respectively. When coding and non-coding regions were considered separately, the combined frequencies of polymorphism per base pair in Groups 1 and 2 were found to be 0.00082 in coding sequence and 0.0027 in non-coding regions.

### Extensive genotyping reveals the frequency of allelic dropout

While screening, we consistently encountered samples for which the sequence traces corresponded to unexpected genotypes. Examples of this can be seen in the genotype ratios in Tables 1 and 2. These samples were of unexpected homozygous genotypes and constituted between 0.7% and 25.9% of the genotyped samples for each amplicon. Where both forward and reverse sequence data was available, it was found to agree. This indicates that the mis-genotyping was not caused by sequencing errors, but instead was due to allele bias during the PCR amplification of heterozygous F1 samples, resulting in the phenomenon known as allelic dropout [12]. Where a sibling group was predicted to contain only heterozygous individuals on the basis of the parental genotypes, as was the case for Group 1 and the cdx4 amplicon, allelic dropout affected both alleles. We calculated the per-genotype allelic dropout rate (i.e. the proportion of mis-genotyped heterozygotes) for amplicons where the progeny should have consisted only of heterozygotes (Group 1 *cdx4*; Group 2 *frizzled7* and *noggin1*). The resulting rates were 0.26, 0.05 and 0.05 for *cdx4*, *frizzled7* and *noggin1* respectively. We also estimated allelic dropout rates for the remaining amplicons, where one class of homozygous genotype was expected in addition to the heterozygous genotype. Assuming allelic dropout to affect both alleles equally, we estimated per-genotype allelic dropout rates ranging from 0.03 (Group 2, *pax2* amplicon 9) to 0.43 (Group 1, *hhex*), with a mean rate of 0.13.

### Sequence comparisons identify both shared and unique polymorphisms in Nigerian and Ivory Coast strains

**Figure 1:**
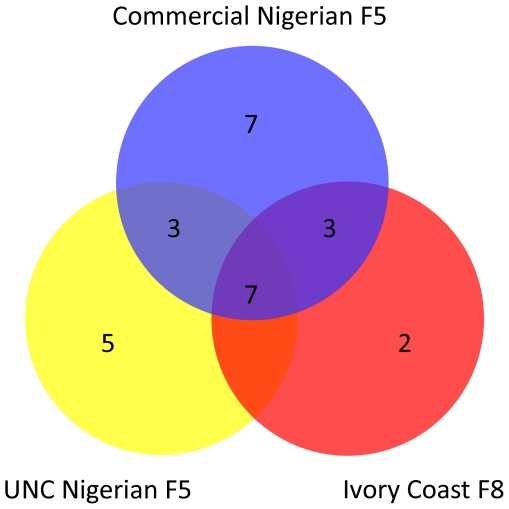
Overview of shared and unique polymorphism amongst sequenced strains. This Venn diagram summarizes the pattern of shared and unique polymorphism between the UNC Nigerian F5, commercial Nigerian F5 and Ivory Coast F8 inbred frogs genotyped for 28 polymorphisms in 12 polymorphic genes.

The *X. tropicalis* most commonly used for research are obtained from a commercial breeding population of animals, originating from Nigeria, that have undergone five generations of sibling mating, followed by indeterminate interbreeding of the stock (L. Northey, Nasco Inc., personal communication; R.M. Harland, http://tropicalis.berkeley.edu/home/genetic_resources/Inbred-strains/Nigerians2/Nigerian.html). The parents of the animals in Groups 1 and 2 described above were our own first-generation lab-bred animals derived from these. Compared with out-bred and wild-caught animals, these Nigerian strain frogs may carry a significantly less diverse pool of naturally-occurring polymorphisms as a result of inbreeding. We decided to examine the polymorphisms carried by a third independent group of Nigerian F5 siblings derived directly from the commercial stock (‘commercial Nigerian F5’), for comparison with our own laboratory stock (‘UNC Nigerian F5’) in order to better assess the degree of genetic diversity present within the Nigerian F5 population. Efforts to identify novel mutants or genetic markers from natural polymorphism pools would likely be more productive if multiple independent strains or wild-caught animals are used, so we also sequenced a second independent strain reported to have been derived from frogs originating from Ivory Coast (‘Ivory Coast F8’). We sequenced our amplicon panel from a set of 29 Nigerian F5 siblings and 22 frogs of the lab-bred Ivory Coast F8 strain. The resulting sequences were aligned as before and screened for both heterozygous and homozygous sequence variation. For the commercial Nigerian F5 frogs, 4,226 bp of coding sequence and 3,853 bp of non-coding sequence were screened. Twenty-one polymorphisms were found in 14 amplicons (of 20 sequenced) (see Table 3), giving frequencies of 0.0012 and 0.0034 polymorphisms per-base pair in coding and non-coding regions respectively. While eleven of the polymorphisms identified (in *cdx4*, *fzd7*, *gata6* amplicon 2a, *hhex*, *oct1*, *pax2* amplicon 2 and *pax8* amplicon 4) were shared with the Group 1 and/or Group 2 UNC Nigerian F5 animals, the remaining ten were not. Similarly, five polymorphisms found in Group 1 and/or Group 2 were not found in the commercial Nigerian F5 siblings. The majority of the polymorphisms found in the commercial Nigerian F5 animals were silent variations in non-coding sequences, but mis-sense and silent SNPs were also found in coding sequences (see Table 3). No further polymorphisms were found when the sequences obtained from these animals were compared with those of Groups 1 and 2. For the Ivory Coast F8 strain, 4,264 bp of coding sequence and 3,740 bp of non-coding sequence were screened. These frogs carried twelve polymorphisms in nine amplicons (of 20 sequenced) (see Table 4). Two of these polymorphisms were unique to the Ivory Coast frogs. From this we calculated polymorphism frequencies of 0.0009 and 0.0021 per-base pair for coding and non-coding regions respectively in the Ivory Coast F8 animals. Alignment of the resulting consensus sequences from the Nigerian and Ivory Coast datasets revealed no additional polymorphisms between the two strains. Note that all of the polymorphisms were found in multiple individuals (Supporting Information S2), with no further variants detected. The genotypes of all the sequenced strains across all amplicons (including those that were not polymorphic) are summarized in Table 5 and Figure 1.

**Table 3:** Polymorphisms identified in commercial Nigerian F5 frogs.

Gene	# base pairs screened	Sequence variants	Class
cdx4	389 bp	a)CTATCaAACAT b)CTATCgAACAT	Silent (non-coding)
eomes	466 bp	a)CCGGAtAACGG b)CCGGAcAACGG	Silent (Asn/Asn88)
frzd7	439 bp	a)CCTCTaTGcACGGA b)CCTCTgTGtACGGA c)CCTCTgTGcACGGA	Silent (Leu/Leu43 and Cys/Cys44)
gata4 (amplicon 1)	402 bp	a)GAATTGCtAGGATG b)GAATTGCcAGGATG	Silent (non-coding)
gata4 (amplicon 2)	378 bp	a)TGAaCCC…GCTaCTT…GGcaccgcgtCT b)TGAgCCC…GCTgCTT…GG--------CT c)TGAgCCC…GCTaCTT…GGcaccgcgtCT	Mis-sense (Ser/Asn140); Mis-sense (Thr/Ala146); Silent (non-coding)
gata6 (amplicon 1)	410 bp	a)CTTGGGcCCCCCCT b)CTTGGG-CCCCCCT	Silent (non-coding)
gata6 (amplicon 2)	456 bp	a)TTGGCCaGCTGG b)TTGGCCtGCTGG	Silent (Pro/Pro278)
gata6 (amplicon 3)	336 bp	a)TTTTTaAATCA b)TTTTTtAATCA	Silent (non-coding)
hhex	431 bp	a)ATTACtTATtAAACA…AACaATT b)ATTACaTATcAAACA…AACtATT c)ATTACaTATtAAACA…AACaATT	Silent (non-coding)
mmp7	355 bp	a)TGGAAtaCAGCC b)TGGAAcgCAGCC	Silent (non-coding)
noggin1	480 bp	b)TGAAcCCCCCCAAT…(CTCCtCTGA) c)TGAA-CCCCCCAAT…(CTCCtCTGA)	Silent (non-coding)
oct1	423 bp	a)CTTTGtttgTATGAT b)CTTTG----TATGAT	Silent (non-coding)
pax2 (amplicon 2)	484 bp	a)(TTTaGTC…)TTA-TTT…GACcCCA c)(TTTaGTC…)TTAtTTT…GACtCCA	Silent (non-coding)
pax8	392 bp	a)TGGAAaGAACA b)TGGAAgGAACA	Silent (non-coding)

Polymorphisms detected by direct sequencing of 29 siblings are summarized. See the legend accompanying Table 1 for a detailed description of the allele notation system used.

**Table 4:** Polymorphisms identified in Ivory Coast F8 frogs.

Gene	# base pairs screened	Sequence variants	Class
frzd7	439 bp	a)CCTCTaTGcACGGA b)CCTCTgTGtACGGA	Silent (Leu/Leu43 and Cys/Cys44)
gata4 (amplicon 2)	378 bp	a)TGAaCCC…(GCTaCTT…GGcaccgcgtCT) c)TGAgCCC…(GCTaCTT…GGcaccgcgtCT)	Mis-sense (Ser/Asn140)
gata6 (amplicon 1)	410 bp	a)CTTGGGcCCCCCCT b)CTTGGG-CCCCCCT	Silent (non-coding)
gata6 (amplicon 2)	456 bp	a)TTGGCCaGCTGG b)TTGGCCtGCTGG	Silent (Pro/Pro278)
hhex	431 bp	a)TACtTATtAAA(…AACaATT) d)TACaTATcAAA(…AACaATT)	Silent (non-coding)
noggin1	480 bp	b)TGAAcCCCCCCAAT…(CTCCtCTGA) c)TGAA-CCCCCCAAT…(CTCCtCTGA)	Silent (non-coding)
pax2 (amplicon 1)	337 bp	a)AGATAcaCACAC b)AGATA–CACAC	Silent (non-coding)
pax2 (amplicon 2)	484 bp	a)(TTTaGTC…)TTA-TTT…GACcCCA c)(TTTaGTC…)TTAtTTT…GACtCCA	Silent (non-coding)
pax6 (amplicon 3)	445 bp	b)TGActaagtcattgaGGA…(GAGgagAAA) c)TGA————GGA…(GAGgagAAA)	Silent (non-coding)

Polymorphisms detected by direct sequencing of 22 individuals of the Ivory Coast strain are summarized. See the legend accompanying Table 1 for a detailed description of the allele notation system used.

**Table 5:** Genotypes of *X.tropicalis* strains for 23 sequenced amplicons.

	Strain			
Gene	UNC Nigerian F5 Group 1	UNC Nigerian F5 Group 2	Nigerian F5 (commercial)	Ivory Coast F8
*cdx4*	a, b	a, b	a, b	a only
*chrd*	a, b	a, b	a only	a only
*eomes*	b only	b only	a, b	a only
*fgfr2*	no polymorphism	no polymorphism	no polymorphism	no polymorphism
*fst*	no polymorphism	no polymorphism	n/s	no polymorphism
*fzd7*	a only	a, b, c	a, b, c	a, b
*gata4* (amplicon 1)	a only	a only	a, b	a only
*gata4* (amplicon 2)	c only	c only	a, b, c	a, c
*gata6* (amplicon 1)	b only	b only	a, b	a, b
*gata6* (amplicon 2)	b only	a, b	a, b	a, b
*gata6* (amplicon 3)	b only	b only	a, b	a only
*hhex*	a, c	a, b, c	a, b, c	a, d
*mmp7*	b only	b only	a, b	b only
*mixer*	no polymorphism	no polymorphism	n/s	n/s
*noggin1*	a, b	a, b	b, c	b, c
*not*	no polymorphism	no polymorphism	n/s	n/s
*oct1*	a, b	a only	a, b	n/s
*pax2* (amplicon 1)	a only	a only	a only	a, b
*pax2* (amplicon 2)	a,c	b, c	a, c	a, c
*pax6* (amplicon 1)	no polymorphism	no polymorphism	no polymorphism	no polymorphism
*pax6* (amplicon 2)	no polymorphism	no polymorphism	no polymorphism	no polymorphism
*pax6* (amplicon 3)	b only	a, b	b only	b, c
*pax8*	a,b	a,b	a, b	b only

For polymorphic amplicons, each allele is represented by a letter code corresponding to those detailed in tables 1, 2, 3, 4. Abbreviation: n/s, not sequenced.

### Frequencies of homozygosity show considerable variation but broadly correspond to expected inbreeding coefficients

The inbreeding coefficient (*F*) is a property of an individual that has undergone a given program of inbreeding and corresponds to the probability that the alleles of a randomly chosen gene are identical by descent from a common ancestral allele [13], [14]. The value of *F* increases with each successive round of inbreeding. In each group of frogs genotyped, we found a range of frequencies of homozygosity at sequenced genes (see Supporting Information S2 for the table of genotypes on which this is based). The distributions of these frequencies and the mean frequency for each strain (UNC Nigerian F5 mean = 0.73 ; commercial Nigerian F5 mean = 0.56; Ivory Coast F8 mean = 0.77) are in broad agreement with the inbreeding coefficients for the appropriate generation of each inbred strain (F5 *F* = 0.67, F8 *F* = 0.83) [13], [15].

### Polymorphism-based phylogenetic trees reflect the origins of laboratory strains

**Figure 2:**
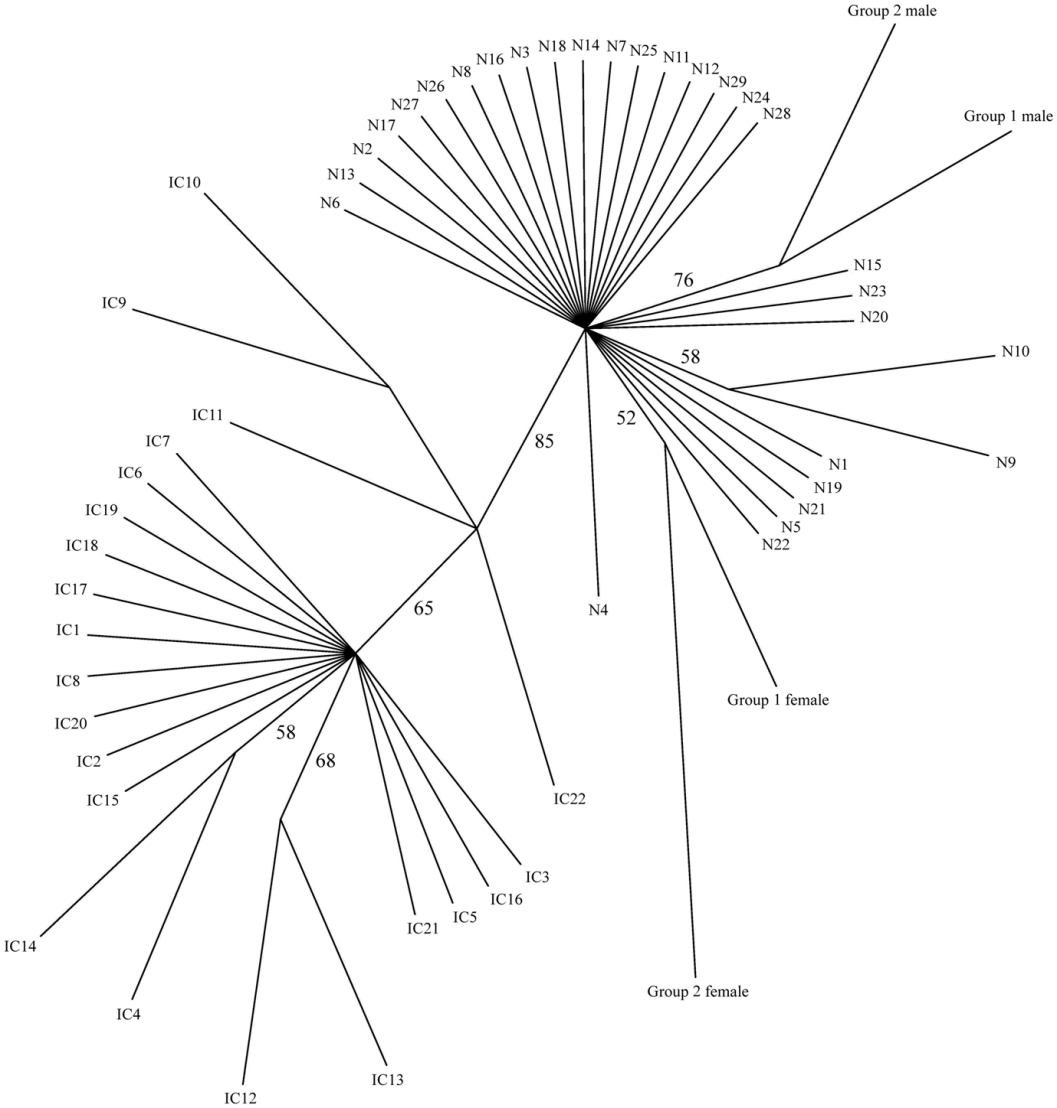
*X. tropicalis* strain phylogeny. A consensus, unrooted, neighbor-joining tree representing the phylogenetic relationships between 55 sequenced individuals is shown. The UNC Nigerian F5 strain is represented by four individuals, the genotyped parents of the group 1 and group 2 animals previously analyzed. The Ivory Coast F8 and commercial Nigerian F5 individuals are labeled IC1-22 and N1-29 respectively. The bootstrap values are shown alongside the branches, indicating the number of times the partition of the individuals into the two sets separated by the branch occurred amongst the 100 trees on which the consensus tree is based.

The degree to which individuals share particular alleles at genotyped loci provides a measure of genetic distance [16], [17]. This can be used to produce phylogenetic trees that interpret polymorphism data and give a visual representation of the genetic relationship between individuals and between strains. Based on inter-individual genetic distances calculated from 18 amplicons genotyped in all sample sets, we performed 100 bootstrap re-samplings and from the resulting datasets produced a consensus, unrooted, neighbor-joining phylogenetic tree showing the relationship between the 55 individuals of the Nigerian (UNC Nigerian F5and commercial Nigerian F5) and Ivory Coast (F8) strains examined in our study (Figure 2). For the purposes of this analysis, the UNC Nigerian F5 strain was represented by the parents of Groups 1 and 2. The resulting tree reflects the known lineages, with individuals primarily grouped into two strain-specific clusters on distinct branches. The clearest distinction, with the strongest support from the bootstrapping analysis, is between frogs originating from Nigeria and those originating from Ivory Coast, while lab-bred UNC Nigerian F5 frogs show the expected close relationship to the commercial Nigerian F5 individuals.

## Discussion

Our screens for sequence variants identified significant naturally occurring polymorphism within laboratory strains of *X. tropicalis*. This provides two important measures of polymorphism. The first is of the frequency at which polymorphism occurs within coding and non-coding nuclear DNA in *X. tropicalis*. The second is of the frequency of polymorphic genes in the genome. Both of these measures are important considerations in the design of reverse genetic screens that utilize these laboratory strains, because interference from naturally-occurring polymorphism poses a challenge when trying to discover rare induced mutations by mismatch based methods or by amplicon resequencing. This challenge can be overcome to some degree by the choice of mutation screening method. For example, CelI screening is an alternative to DHPLC that is still able to detect most induced mutations in the presence of polymorphism. While CelI cleaves mismatched DNA at sites of naturally-occurring polymorphism, cleavage at additional mismatches arising from mutation results in a detectable change in the ‘fingerprint’ of a mutant sample. In amplicon re-sequencing screens, polymorphism can be overcome through the use of analysis software to distinguish between polymorphisms and induced mutations [3]. In future, laboratory strains that have been inbred through further generations will eliminate this issue and be invaluable for genetic screening.

The frequency of sequence polymorphism in a laboratory strain is determined by the frequency of polymorphism in the founders of the strain, the frequency of spontaneous mutation and the degree to which the strain has undergone inbreeding. It is important to note that the frequency of polymorphism detected in this study constitutes a minimal estimate of the actual frequency of polymorphism in the screened genes, as the amplicons analyzed by re-sequencing encompass only a part of each gene and other regions may harbor polymorphism. The actual frequency of polymorphic genes amongst those screened may therefore be higher, but not lower, than we report here. It is important to compare our results with the expected inbreeding coefficient, a measure of the percentage of genes or loci that are expected to be identical by descent and therefore non-polymorphic or ‘fixed’ in the genome of an individual derived from a given number of generations of sibling mating. The frequency of homozygosity in individuals of each strain was found to agree quite well with predicted frequencies. This is somewhat surprising since Wright's inbreeding coefficient is calculated based on the assumption that more than one allele is present for every gene in the founders, i.e. no homozygous (or non-polymorphic) genes. If the accepted lineages are assumed to be accurate, this would suggest that wild populations from which the founders of these strains were collected are very genetically diverse. Alternatively, the frequency of homozygosity may have been reduced at some point in the lineages through inadvertent outcrossing. The frequency of homozygosity may also be reduced due to disproportionate selection of unfixed genes for screening. It should be noted that this type of sampling bias, which may result from our decision to focus on selected groups of genes involved in the control of embryonic development, could also affect our estimates of polymorphism frequencies at the DNA sequence level. While we hope that our data will be of particular relevance to investigators who wish to conduct genetic screens for natural variants or induced mutations in developmental genes, genome-wide polymorphism discovery efforts will be required to determine whether our polymorphism frequency estimates hold true across the whole genome.

Collectively, the strains we have analyzed carry a relatively low frequency of polymorphism compared to that found in mouse. A study of the genetic diversity between six wild-derived *Mus musculus domesticus* inbred strains found polymorphism frequencies (for SNPs and indels) of 0.0077/bp and 0.0188/bp for coding and non-coding regions respectively [18]. The mouse is the most genetically diverse mammalian species known, with approximately one order of magnitude more variant positions than found in human genomes [19]. This peculiarly high level of diversity in mouse, coupled with unexpected relatedness between the wild populations of *X. tropicalis* in Nigeria and Ivory Coast from which the founders of the sequenced strains were collected, probably underlies the difference between *X. tropicalis* and *M.m.domesticus* polymorphism frequencies. Sequence analysis of other strains of *X. tropicalis*, in particular the TGA Ivory Coast strain, may uncover greater genetic diversity that could enhance ecotilling efforts.

The sequence polymorphism we identified is significant in at least three respects. Firstly, the degree to which the collections of polymorphisms differed amongst Nigerian strain animals suggests that existing stocks are likely to harbor a diverse pool of sequence variants. The potential utility of some of these variants is shown by the skewed distribution of certain alleles which, although not deleterious themselves, nevertheless appear to be linked to deleterious alleles at syntenic loci. Ecotilling in diverse groups of Nigerian strain animals may therefore be a means of isolating functionally informative alleles, particularly if performed on sample sets that allow non-Mendelian distributions to be detected. The second respect in which the observed polymorphism is significant relates to the design of tilling screens in *X. tropicalis*, as discussed above. Finally, the polymorphisms identified in our study have the potential to be used as markers for genetic mapping studies in *X. tropicalis* and our data suggests that more such polymorphisms are likely to exist between laboratory strains. A sufficiently large collection of SNPs, combined with existing methods for high-throughput SNP genotyping, would be a valuable tool for mapping mutations isolated in phenotype-based screens.

Important for mutation mapping is the observation that our genotyping data contained a significant frequency of allelic dropout. This phenomenon has been observed in numerous other studies and the frequencies we determined fall within the range reported by other investigators [12]. It is often presumed to arise in part from sequence heterogeneity at PCR primer sites causing amplification bias [12]. However, we detected no bias towards dropout of particular alleles in which primer site variants might exist. It has been suggested that an alternative cause of allelic dropout is the low concentration of primer binding sites in genomic DNA, leading to amplification bias due to the stochastic nature of PCR [12], [20]. The yield of genomic DNA from tadpole tissue is typically low and so this may be a contributing factor to the allelic dropout we observed. Allelic dropout in genotype-based reverse genetic screens could result in a failure to identify a subset of induced mutations, but it is likely to be more problematic when mapping mutations uncovered in phenotype-based forward genetic screens. Mapping typically involves PCR amplification and genotyping of hundreds or thousands of microsatellite markers in order to calculate the recombination frequencies on which linkage map positions are based. Mis-genotyping of samples at rates similar to those occurring in our study could significantly alter the calculated map position of a mutated locus relative to linked markers. This under-appreciated effect has been demonstrated to lead to an inflation of map distances in the context of high-resolution maps consisting of many markers [21], [22], [23]. In simpler two-point and three-point mapping of mutations, genotyping errors resulting from allelic dropout lead to underestimation of map distances because of the mis-genotyping of recombinants. Genotyping samples in duplicate can overcome the problem but dramatically increases the labor required, complicating the analysis by requiring the genotypes of duplicates to be cross-referenced against one another. An alternative mapping method – bulked segregant analysis of random amplified polymorphic DNA (RAPD) markers - is based on the amplification of polymorphic markers from haploid individuals and is not subject to allelic dropout [24]. This method produced the first genetic map in zebrafish [25], [26], [27] and could be used in *X. tropicalis* to map mutations relative to the existing SSR marker set or to polymorphisms of the types identified in our study. Further ecotilling could contribute many more polymorphisms for mapping, in addition to uncovering valuable alleles for functional studies. In these respects, ecotilling complements other types of genetic screening (e.g. phenotype-based mutation screens, tilling etc.) and may therefore make an important contribution to the future development of *X. tropicalis* as a useful genetic model system.

## Methods

### Ethics Statement

All animal experiments were performed in accordance with protocols approved by the Institutional Animal Care and Use Committee of the University of North Carolina at Chapel Hill (IACUC approval # 07-289.0-C).

### Breeding, tissue sampling and DNA preparation

*X. tropicalis* used were F5 Nigerian-strain frogs purchased from Nasco International Inc. (Fort Atkinson, WI), a lab-bred mixed-lineage stock (‘UNC Nigerian F5’) derived from these, or F8 Ivory Coast frogs derived from the American Ivory Coast line established at the University of Virginia, USA and obtained from L.B. Zimmerman (National Institute for Medical Research, UK). Natural mating, embryo collection and tadpole husbandry were performed as described previously [28]. Whole tadpole lysis was carried out using a lysis method described previously [29]. Tissue samples from juvenile frogs were taken from trunk body wall muscle following euthanasia. Tissue samples from adult frogs were obtained by toe-clipping following anesthesia in a 0.025% (w/v) solution of Tricaine (Sigma-Aldrich Corp.). Genomic DNA was purified from tissue samples as described by Wienholds and co-workers [30].

### Sequence variant detection by sequencing

PCR primer sequences were based on the JGI *X. tropicalis* genome sequence v4.1 (http://genome.jgi-psf.org/Xentr4/Xentr4.home.html). See Supporting Information S1 for primer sequences. PCR and sequencing was carried out as described by Goda et al. (2006). Each amplicon was sequenced in both directions for each sample. For each amplicon, sequence traces were aligned and screened for heterozygous bases using CodonCode Aligner (CodonCode Corp., Dedham, MA). F1 genotypes were confirmed by visual analysis of individual sequence traces. For parental genotyping, two independent PCR reactions were carried out per parent per amplicon and sequenced in both directions. Individual sequence traces were visualized using FinchTV v1.4 software (GeoSpiza Inc., Seattle, WA). Sequencher v4.8 software (Gene Codes Corp., Ann Arbor, MI) was used for inter-strain comparisons of amplicon consensus sequences. Raw sequence data are available at Dryad (http://datadryad.org): doi:10.5061/dryad.742j4 . The variants reported have been deposited in the dbSNP database (http://www.ncbi.nlm.nih.gov/projects/SNP/).

### Statistical analysis

The statistical significance of the observed genotype distributions was determined by the Chi-square test with Yates' correction for continuity.

### Phylogenetics

The 100 bootstrap datasets were produced using Seqboot (PHYLIP software package) [31]. Genetic distance values for each dataset were calculated as 1−P_S_, where P_S_ corresponded to the proportion of shared alleles, using ‘Individual to Individual Genetic Distance Calculator’ [32] to generate 100 distance matrices. Unrooted trees were generated using NEIGHBOR (PHYLIP) and the consensus tree was generated using CONSENSE (PHYLIP).

## Supporting Information

Supporting Information S1 Sequences of oligonucleotide primer pairs used for PCR.
(DOC)

Supporting Information S2 Table of individual genotypes at 17 sequenced amplicons. Genotypes of sequenced individuals from UNC Nigerian F5 (Group 1/Group 2), commercial Nigerian F5 (N1-N29) and Ivory Coast F8 (IC1-IC22) strains at the following amplicons: 1) *cdx4*; 2) *chordin*; 3) *frizzled7*; 4) *gata4* amplicon 1+ amplicon 2; 5) *gata6* amplicon 1; 6) *gata6* amplicon 2; 7) *hhex*; 8) *mmp7*; 9) *noggin1*; 10) *pax2* amplicon 1; 11) *pax2* amplicon 2; 12) *pax6* amplicon 1; 13) *pax6* amplicon 2; 14) *pax6* amplicon 3; 15) *pax8*; 16) *eomes*; 17) *gata6* amplicon 3.
(DOC)

## References

[1] Showell C, Conlon FL (2007). Decoding development in Xenopus tropicalis.. Genesis.

[2] Hellsten U, Harland RM, Gilchrist MJ, Hendrix D, Jurka J (2010). The genome of the Western clawed frog Xenopus tropicalis.. Science.

[3] Goda T, Abu-Daya A, Carruthers S, Clark MD, Stemple DL (2006). Genetic screens for mutations affecting development of Xenopus tropicalis.. PLoS Genet.

[4] Grammer TC, Khokha MK, Lane MA, Lam K, Harland RM (2005). Identification of mutants in inbred Xenopus tropicalis.. Mech Dev.

[5] Cargill M, Altshuler D, Ireland J, Sklar P, Ardlie K (1999). Characterization of single-nucleotide polymorphisms in coding regions of human genes.. Nat Genet.

[6] Comai L, Young K, Till BJ, Reynolds SH, Greene EA (2004). Efficient discovery of DNA polymorphisms in natural populations by Ecotilling.. Plant J.

[7] Barkley NA, Wang ML (2008). Application of TILLING and EcoTILLING as Reverse Genetic Approaches to Elucidate the Function of Genes in Plants and Animals.. Curr Genomics.

[8] Gilchrist EJ, Haughn GW, Ying CC, Otto SP, Zhuang J (2006). Use of Ecotilling as an efficient SNP discovery tool to survey genetic variation in wild populations of Populus trichocarpa.. Mol Ecol.

[9] Till BJ, Zerr T, Bowers E, Greene EA, Comai L (2006). High-throughput discovery of rare human nucleotide polymorphisms by Ecotilling.. Nucleic Acids Res.

[10] Xu Z, Gutierrez L, Hitchens M, Scherer S, Sater AK (2008). Distribution of polymorphic and non-polymorphic microsatellite repeats in Xenopus tropicalis.. Bioinform Biol Insights.

[11] Wells DE, Gutierrez L, Xu Z, Krylov V, Macha J A genetic map of Xenopus tropicalis.. Dev Biol.

[12] Pompanon F, Bonin A, Bellemain E, Taberlet P (2005). Genotyping errors: causes, consequences and solutions.. Nat Rev Genet.

[13] Wright S (1921). Systems of Mating. II. the Effects of Inbreeding on the Genetic Composition of a Population.. Genetics.

[14] Wright S (1922). Coefficients of Inbreeding and Relationship.. The American Naturalist.

[15] Kurosawa T (2001). Inbred Animal Strains..

[16] Ben-Ari G, Zenvirth D, Sherman A, Simchen G, Lavi U (2005). Application of SNPs for assessing biodiversity and phylogeny among yeast strains.. Heredity.

[17] Bowcock AM, Ruiz-Linares A, Tomfohrde J, Minch E, Kidd JR (1994). High resolution of human evolutionary trees with polymorphic microsatellites.. Nature.

[18] Ideraabdullah FY, de la Casa-Esperon E, Bell TA, Detwiler DA, Magnuson T (2004). Genetic and haplotype diversity among wild-derived mouse inbred strains.. Genome Res.

[19] Sachidanandam R, Weissman D, Schmidt SC, Kakol JM, Stein LD (2001). A map of human genome sequence variation containing 1.42 million single nucleotide polymorphisms.. Nature.

[20] Taberlet P, Griffin S, Goossens B, Questiau S, Manceau V (1996). Reliable genotyping of samples with very low DNA quantities using PCR.. Nucleic Acids Res.

[21] Buetow KH (1991). Influence of aberrant observations on high-resolution linkage analysis outcomes.. Am J Hum Genet.

[22] Goldstein DR, Zhao H, Speed TP (1997). The effects of genotyping errors and interference on estimation of genetic distance.. Hum Hered.

[23] Hackett CA, Broadfoot LB (2003). Effects of genotyping errors, missing values and segregation distortion in molecular marker data on the construction of linkage maps.. Heredity.

[24] Williams JG, Kubelik AR, Livak KJ, Rafalski JA, Tingey SV (1990). DNA polymorphisms amplified by arbitrary primers are useful as genetic markers.. Nucleic Acids Res.

[25] Postlethwait JH, Talbot WS (1997). Zebrafish genomics: from mutants to genes.. Trends Genet.

[26] Johnson SL, Midson CN, Ballinger EW, Postlethwait JH (1994). Identification of RAPD primers that reveal extensive polymorphisms between laboratory strains of zebrafish.. Genomics.

[27] Postlethwait JH, Johnson SL, Midson CN, Talbot WS, Gates M (1994). A genetic linkage map for the zebrafish.. Science.

[28] Showell C, Conlon FL (2009). Natural Mating and Tadpole Husbandry in the Western Clawed Frog *Xenopus tropicalis*.. Cold Spring Harb Protoc.

[29] Showell C, Conlon FL (2009). Tissue sampling and genomic DNA purification from the western clawed frog Xenopus tropicalis.. Cold Spring Harb Protoc.

[30] Wienholds E, van Eeden F, Kosters M, Mudde J, Plasterk RH (2003). Efficient target-selected mutagenesis in zebrafish.. Genome Res.

[31] Felsenstein J (1989). PHYLIP - Phylogeny Inference Package (Version 3.2).. Cladistics.

[32] Brzustowski J http://www2.biology.ualberta.ca/jbrzusto/sharedst.php.

