# Supplementary material for: A Comparative Survey of the Frequency and Distribution of Polymorphism in the Genome of Xenopus tropicalis
Source: PLoS One. 2011 Aug 4;6(8):e22392. doi: 10.1371/journal.pone.0022392 (PMC3150332; doi:10.1371/journal.pone.0022392)
Supplement: Supporting Information S1 — Sequences of oligonucleotide primer pairs used for PCR. (DOC) [file pone.0022392.s001.doc]

**SUPPORTING INFORMATION S1**

Sequencing amplicon PCR primers

**caudal type homeobox 4 (cdx4)** 5’-CAGAGCAGAGAGTCGAGGCAAATG-3’,

5’-GCGATACAAATGACCTCTG-3’

**chordin (chrd),** 5’-GACCCAGAGAGCCAGAGAC-3’,

5’-CTGATGACTGCCCTAGCTG-3’

**eomesodermin (eomes)** 5’-GCCATTTCCTATGATTCAG-3’,

5’-GCTGTAGGTAGTAGCGCTCAG-3’

**fibroblast growth factor receptor 2 (fgfr2)** 5’-GATATTTCTCGCATACAGATGG-3’,

5’-GCCAGTTACATACACCTCCTG-3’

**follistatin (fst)** 5’-GACATGCTGCTGAGATATGG-3’,

5’-CATCACAGCAATCTAATGTGG-3’

**frizzled homolog 7 (fzd7)** 5’-AGGAGAATTAGCTGGTTTGG-3’,

5’-CACTGGAAGCCGAACTTG-3’

**gata binding protein 4 (gata4), amplicon 1** 5’-ACTGAAGGACGCTGAAACTC-3’,

5’-GTACTGCTCTCTCCCATTCG-3’

**gata4, amplicon 2** 5’-GGCTCATCTCAACAAGGAAG-3’,

5’-CCAATAAACCCAACATTTCC-3’

**gata binding protein 6 (gata6), amplicon 1** 5’-AATGGGAAAGTGCTTTGTG-3’,

5’-GCTTTACCAGGTCTCATTGG-3’

**gata6, amplicon 2** 5’-CTATGTGCCCACTAGCAGAG-3’,

5’-TGGATTCCCAAATTACCAAC-3’

**gata6, amplicon 3** 5’-AAGTTGTTGGTACGGACAGG-3’,

5’- CCCGTGTATTTAGGTGATTG-3’

**hematopoietically expressed homeobox (hhex)** 5’-TTCCCAAGGAGGTAACCAG-3’,

5’-CACTGAGAAACTGGCAGATG-3’

**matrix metalloprotease 7 (mmp7)** 5’-GCCTTAAGAACAAATTGCAC-3’,

5’-AGTTCCTGCCCTATGTCATC-3’

**mix-like endodermal regulator (mixer)** 5’-CCCAGAGAGGTATAAGAAGG-3’,

5’-GGAGTGCAGGGCATATAAAC-3’

**noggin 1** 5’-CACTTGTCTGCAATGAGAGG-3’,

5’-TCCTGAGTTTCTTGCTCAGTC-3’

**notochord homeobox (not)** 5’-CTGTCTTTCCTGCATTTGG-3’,

5’-GCTACATTTGATACTTGATTTGC-3’

**paired box 2 (pax2), amplicon 1** 5’-GATGCTGATCACATTTCTGC-3’,

5’- TTGACTCCACGTGGTTTATG-3’

**pax2, amplicon 2** 5’-AGCTGTTCTTGACCTTAGTCG -3’,

5’-TGGTTGCTGACATTTATGAG-3’

**paired box 6 (pax6), amplicon 1** 5’-AAAGTCCCAGTGCTGGATAG-3’,

5’-GCTCACAATATGTTCAAACACTACC-3’

**pax6, amplicon 2** 5’-TATGAAGATTCGGTGGTAGC-3’, 5’-GTGTCGCTATCGATCTCTCC-3’

**pax6, amplicon 3** 5’-GGTCATGCAATAACAGTTGC-3’, 5’-ATTCTCATTGGCCAACTCTC-3’

**paired box 8 (pax8)** 5’-GATGGGTCTGCTATGCTACC-3’,

5’-GCACACATAAATGTGGGAATAC-3’

**pou class 2 homeobox 1 (pou2f1; oct1)** 5’-TTGGCTCAATGTTATCCCTAC-3’,

5’-AATAAGCACCTGGTTTGAG-3’
