# Supplementary material for: A Comparative Survey of the Frequency and Distribution of Polymorphism in the Genome of Xenopus tropicalis
Source: PLoS One. 2011 Aug 4;6(8):e22392. doi: 10.1371/journal.pone.0022392 (PMC3150332; doi:10.1371/journal.pone.0022392)
Supplement: Supporting Information S2 — Table of individual genotypes at 17 sequenced amplicons. Genotypes of sequenced individuals from UNC Nigerian F5 (Group 1/Group 2), commercial Nigerian F5 (N1-N29) and Ivory Coast F8 (IC1-IC22) strains at the following amplicons: 1) cdx4; 2) chordin; 3) frizzled7; 4) gata4 amplicon 1+ amplicon 2; 5) gata6 amplicon 1; 6) gata6 amplicon 2; 7) hhex; 8) mmp7; 9) noggin1; 10) pax2 amplicon 1; 11) pax2 amplicon 2; 12) pax6 amplicon 1; 13) pax6 amplicon 2; 14) pax6 amplicon 3; 15) pax8; 16) eomes; 17) gata6 amplicon 3. (DOC) [file pone.0022392.s002.doc]

| **Amplicon**  **Frog** | **1** | **2** | **3** | **4** | **5** | **6** | **7** | **8** | **9** | **10** | **11** | **12** | **13** | **14** | **15** | **16** | **17** |
| --- | --- | --- | --- | --- | --- | --- | --- | --- | --- | --- | --- | --- | --- | --- | --- | --- | --- |
| **Group 4 male** | a a | a b | a a | a a | b b | b b | a c | b b | a b | a a | c c | a a | a a | b b | a b | b b | b b |
| **Group 4 female** | b b | a a | a a | a a | b b | b b | a a | b b | a a | a a | a c | a a | a a | b b | b b | b b | b b |
| **Group 5 male** | a a | a a | b c | a a | b b | b b | a c | b b | b b | a a | c c | a a | a a | b b | a b | b b | b b |
| **Group 5 female** | a b | a b | a a | a a | b b | a b | a b | b b | a a | a a | b c | a a | a a | b a | b b | b b | b b |
| **N1** | a a | a a | a b | a c | b a | a b | a b | - - | - - | a a | a a | a a | a a | b b | b b | b b | a b |
| **N2** | a a | a a | a a | a c | b a | a b | a c | b b | a a | a a | a c | a a | a a | b b | a a | b b | a b |
| **N3** | a a | a a | a b | a b | b a | a b | - - | b b | c c | a a | a a | a a | a a | b b | b b | a b | a b |
| **N4** | a a | a a | a c | a b | b b | b b | a c | a a | a c | a a | a c | a a | a a | b b | b b | b b | b b |
| **N5** | a b | a a | a b | a b | - - | a b | c c | a a | - - | a a | c c | a a | a a | b b | a b | b b | a b |
| **N6** | a a | a a | a a | a b | b a | a b | c c | b b | a a | a a | c c | a a | a a | b b | b b | b b | a b |
| **N7** | a a | a a | a a | a c | b a | a b | a c | b b | a a | a a | a c | a a | a a | b b | b b | a b | a b |
| **N8** | a b | a a | a c | a b | b a | b b | a b | b b | c c | a a | a c | a a | a a | b b | a b | a b | a b |
| **N9** | a b | a a | b c | a b | b a | a b | a c | b b | a c | a a | c c | a a | a a | b b | b b | a b | a b |
| **N10** | a b | a a | b c | a b | b a | a b | - - | b b | a c | a a | c c | a a | a a | b b | a a | a b | a b |
| **N11** | a b | a a | - - | a b | b b | b b | b c | b b | c c | a a | a c | a a | a a | b b | b b | a b | b b |
| **N12** | a b | a a | - - | a c | b b | b b | - - | a a | a a | a a | a a | a a | a a | b b | b b | a b | b b |
| **N13** | a b | a a | - - | a b | b a | a b | c c | a a | a a | a a | a c | a a | a a | b b | b b | b b | a b |
| **N14** | a a | a a | a c | a b | b b | b b | a b | a b | a c | a a | a a | a a | a a | b b | a a | b b | b b |
| **N15** | a b | a a | - - | a b | b a | a b | c b | a b | c c | a a | a c | a a | a a | b b | a b | a b | a b |
| **N16** | a b | a a | b c | a c | b b | b b | a b | a a | a c | a a | a a | a a | a a | b b | b b | b b | b b |
| **N17** | a a | a a | a a | a c | b b | b b | c b | a b | a c | a a | a c | a a | a a | b b | b b | b b | b b |
| **N18** | a a | a a | b c | a c | b a | a b | c b | a a | a a | a a | a c | a a | a a | b b | a b | b b | a b |
| **N19** | a b | a a | a b | a c | b a | a b | c b | b b | a c | a a | a c | a a | a a | b b | a b | b b | a b |
| **N20** | a a | a a | a c | a c | b a | a b | c c | - - | c c | a a | c c | a a | a a | b b | b b | a b | a a |
| **N21** | a a | a a | b c | a c | b b | b b | a b | b b | a c | a a | a c | a a | a a | b b | b b | a b | b b |
| **N22** | a b | a a | b c | a b | b b | b b | a b | a a | c c | a a | a c | a a | a a | b b | a a | b b | b b |
| **N23** | a b | a a | a c | a b | b a | a b | a b | b b | a c | a a | a a | a a | a a | b b | b b | a b | a b |
| **N24** | - - | a a | - - | a b | b b | b b | c b | b b | a c | a a | a c | a a | a a | b b | a b | a b | a b |
| **N25** | a b | a a | - - | a b | b b | b b | a b | a a | a a | a a | a a | a a | a a | b b | a b | a b | a b |
| **N26** | a a | a a | - - | a c | b a | a b | a b | a a | a a | a a | a a | a a | a a | b b | b b | a b | a b |
| **N27** | a b | a a | - - | a b | b a | a b | c b | a a | a a | a a | a c | a a | a a | b b | a b | a b | a a |
| **N28** | a b | a a | a b | a c | b a | a b | c b | a a | a a | a a | a a | a a | a a | b b | a b | a b | a a |
| **N29** | a b | a a | a a | a c | b a | a b | a b | a b | c c | a a | a a | a a | a a | b b | a a | a b | a a |
| **IC1** | a a | a a | a a | a c | a a | a a | a a | b b | a c | a b | c c | a a | a a | c c | b b | a a | a a |
| **IC2** | a a | a a | a a | a c | a a | a a | a a | b b | c c | a b | c c | a a | a a | c c | b b | a a | a a |
| **IC3** | a a | a a | a a | a a | a a | a a | a a | b b | a c | b b | c c | a a | a a | c c | b b | a a | a a |
| **IC4** | a a | a a | a a | a a | a a | a a | a a | b b | c c | a b | c c | a a | a a | b c | b b | a a | a a |
| **IC5** | a a | a a | a a | a a | a a | a a | a a | b b | a c | a b | c c | a a | a a | b c | b b | a a | a a |
| **IC6** | a a | a a | a b | a c | a a | a a | a a | b b | a c | a b | c c | a a | a a | b c | b b | a a | a a |
| **IC7** | a a | a a | - - | a c | a a | a a | a a | b b | c c | a b | c c | a a | a a | c c | b b | a a | a a |
| **IC8** | a a | a a | - - | a c | a a | a a | a a | b b | a c | b b | c c | a a | a a | b c | b b | a a | a a |
| **IC9** | a a | a a | a b | a c | b a | a b | a b | b b | a c | a b | a c | a a | a a | c c | b b | a a | a a |
| **IC10** | a a | a a | a b | a c | b a | a b | a b | b b | a c | a b | a c | a a | a a | b c | b b | a a | a a |
| **IC11** | a a | a a | a a | a c | - - | a b | - - | b b | a c | a b | a c | a a | a a | c c | b b | a a | a a |
| **IC12** | a a | a a | a a | a a | a a | a a | a a | b b | c c | b b | c c | a a | a a | c c | b b | a a | a a |
| **IC13** | a a | a a | a a | a a | a a | a a | a a | b b | c c | b b | c c | a a | a a | c c | b b | a a | a a |
| **IC14** | a a | a a | a a | a a | a a | a a | a a | b b | c c | a b | c c | a a | a a | b c | b b | a a | a a |
| **IC15** | a a | a a | a b | a a | a a | a a | a a | b b | a a | a b | c c | a a | a a | c c | b b | a a | a a |
| **IC16** | a a | a a | a b | a a | a a | a a | a a | b b | a a | a b | c c | a a | a a | b c | b b | a a | a a |
| **IC17** | a a | a a | a b | a c | a a | a a | a a | b b | a a | a b | c c | a a | a a | b c | b b | a a | a a |
| **IC18** | a a | a a | a b | a c | a a | a a | a a | b b | a c | b b | c c | a a | a a | b c | b b | a a | a a |
| **IC19** | a a | a a | - - | a a | a a | a a | a a | b b | a a | b b | c c | a a | a a | c c | b b | a a | a a |
| **IC20** | a a | a a | - - | a a | a a | a a | a a | b b | a a | a b | c c | a a | a a | b c | b b | a a | a a |
| **IC21** | a a | a a | - - | a c | a a | a b | a a | b b | a c | b b | a c | a a | a a | b c | b b | a a | a a |
| **IC22** | a a | a a | a a | a c | a a | a b | a b | b b | a a | a b | a c | a a | a a | c c | b b | a a | a a |
